# The Town Planning Acts, 1909 and 1919

**Published:** 1923-01

**Authors:** Edmund R. Abbott

**Affiliations:** Legal Member of the Town Planning Institute


### THE TOWN PLANNING ACTS, 1909 AND 1919.

### SOME DIFFICULTIES.

By EDMUND R. ABBOTT, Legal Member of the Town Planning Institute.

It is now 13 years since the first Town Planning Act was passed, and opportunities have been given to consider its effect and some of the difficulties which have arisen and are still arising.

There can be no doubt that Town Planning is one of the most important branches of Local Government from the point of view of Public Health, and great advantages have already accrued. Perhaps the outstanding feature is the limitation of the number of buildings to a given area. When we think of the old method of development, when working-class houses were built sometimes 40 to the acre, and find that in practically all the state-aided housing schemes the density is limited to 12 to the acre, (the standard fixed by the earliest Town Planning schemes), the effect of the provision in the Act is seen at once. With all the criticism of these housing schemes the principle of a layout on the above lines has been universally praised, and had this been adopted during the greater part of the last century we should probably now be incurring far less expenditure on public health remedial measures.

The Act of 1919 amended several of the provisions of the Act of 1909 by simplifying procedure; it abolished the necessity of obtaining the authority of the Minister of Health to prepare a scheme, and also of schemes being laid upon the Tables of both Houses of Parliament for some weeks.

There are, however, still one or two difficulties which remain, and certainly one which can only be remedied by the further intervention of Parliament—this is the inability, generally speaking, to deal in Town Planning schemes with built-up areas.

The present position is that Town Improvements, including the abolition of slums, can only be dealt with as to the first by local Acts (which apply generally only in large towns), or by means of the procedure under the Public Health Acts, and in the case of slums by schemes under the Housing Acts.

The procedure under the Public Health Acts for the improvement and widening of streets, in the absence of agreement with all the owners of property concerned, involves the promotion of a Provisional Order confirmed by Parliament. The promotion can only be undertaken once a year, and is a somewhat cumbrous matter, involving a local inquiry by the Ministry of Health, and persons interested have the right to oppose the confirming Bill in both Houses of Parliament.

It will be remembered that the Royal Commission as to Housing in Scotland recommended the abolition of the restriction of schemes to land in course of development or likely to be used for building purposes, and only permitting the inclusion in schemes of land already built upon when it appears to the Minister of Health that "a piece of land not likely to be used for building purposes" is so situated with respect to any land likely to be used for building purposes that it ought to be included in any Town Planning scheme made with respect to the last-mentioned land. This has created an unnecessary difficulty for local authorities, and when the amending Act of 1919 was before Parliament efforts were made to sweep away the restriction. It was, however, objected to by some of the large City Corporations, who had already power under Local Acts for dealing with City re-planning. Owing to the Town Planning clauses being dealt with in a Bill which primarily dealt with housing, which was a matter of extreme urgency, it was not possible to press the entire omission of the restriction, and only a slight modification was secured; thus the main difficulty remains. The Minister of Health, therefore, has to decide whether the built-up area proposed to be included does in fact come within the provision of Section 54, sub-section 3, of the Act of 1909 as amended by the third schedule to the Act of 1919.

The effect of the restriction has been largely felt in connection with the construction of arterial roads.

The second difficulty is the question of the area of schemes. Local authorities in many cases have been disposed to confine their schemes to their own districts, and landowners have objected that their protection frequently depends very much upon the inclusion of adjacent lands which, because they are outside the area of the proposed scheme, are left quite uncontrolled, and the lands included are thereby damaged.

Again, this question of area has caused difficulty in the laying out of the lines of arterial roads. An arterial road by its very name is obviously one which must extend for many miles, and will traverse several districts. If one authority prepares a scheme and the adjoining authority does not, the site of the road may be blocked by the erection of buildings which will ultimately have to be acquired at heavy expense.

This difficulty has become very real, especially in view of the large number of new roads and improvements of existing roads which are now being made and carried out in connection with schemes for the relief of unemployment. The Minister of Health is therefore encouraging local authorities to enter into joint arrangements for the preparation of schemes. In some cases this has been done by a Joint Committee with delegation of powers from the local authorities, as in West Middlesex, where some 20 or more borough and district councils have combined, or as in the case of Manchester and the surrounding districts, where some 40 authorities have formed a Joint Advisory Committee for the purpose of co-ordinating the various schemes.

Having regard to the fact that in all boroughs and urban districts with a population of over 20,000 at the last census the authorities are required within the next three years to prepare Town Planning schemes, it will probably be found imperative that the schemes should be jointly prepared or co-ordinated.

The general lines of the schemes over these wide areas will be laid down in outline, leaving the local details to be filled in by the local authorities; and the completed schemes can be either joint schemes or several separate schemes all co-ordinated so as to fit in one with another.

The third and last difficulty to which it is proposed to call attention is the lack of any simple provision for the modification or alteration of existing schemes. The only method at present is by the preparation of a new scheme. There are, of course, difficulties with regard to questions of compensation and betterment, but it does seem desirable to invest the Minister of Health with power by order to vary or modify schemes without involving the local authority in the somewhat lengthy and expensive procedure of the preparation of a new scheme with a new series of maps and other details.

